# Impact of SARS-CoV-2 (Coronavirus) Pandemic on Public Mental Health

**DOI:** 10.3389/fpubh.2020.00292

**Published:** 2020-06-23

**Authors:** Bilal Javed, Abdullah Sarwer, Erik B. Soto, Zia-ur-Rehman Mashwani

**Affiliations:** ^1^Faculty of Sciences, PMAS Arid Agriculture University, Rawalpindi, Pakistan; ^2^Roy and Diana Vagelos Laboratories, Department of Chemistry, University of Pennsylvania, Philadelphia, PA, United States; ^3^Nawaz Sharif Medical College, University of Gujrat, Gujrat, Pakistan; ^4^Allama Iqbal Memorial Teaching Hospital, Sialkot, Pakistan; ^5^Graduate School of Public Health, University of Pittsburgh, Pittsburgh, PA, United States

**Keywords:** SARS-CoV-2, COVID-19, pandemic, mental health, stigma

## Introduction

By way of media reporting, people are being well-informed about the physical effects that the coronavirus can have on the body and what they should do if symptoms appear. However, the effects of this pandemic on one's mental health have not been studied at length and are still unknown. As all efforts are focused on understanding the clinical features, epidemiology, transmission patterns, and management of the COVID-19 outbreak, there has been very little concern expressed over the effects on one's mental health and on strategies to prevent stigmatization (1). People's behavior during the outbreak may greatly affect the pandemic's dynamic by altering the severity, transmission, disease flow, and morbidity and mortality rates worldwide. The present situation requires raising awareness among the public that can be helpful to deal with this unprecedented situation (2). This study provides a detailed overview of the effects of the COVID-19 outbreak on the mental health of individuals.

A pandemic is not just a medical phenomenon, it affects individuals and society and causes disruption, anxiety, stress, stigma, and xenophobia (1). The behavior of an individual as a unit of society or a community has marked effects on the dynamics of a pandemic that involves the level of severity, degree of flow, and aftereffects (3). Rapid human-to-human transmission of SARS-CoV-2 resulted in the enforcement of regional lockdowns to stem the further spread of the disease (4). Isolation, social distancing, closure of educational institutes, workplaces, places of worship, and entertainment hubs confined people to stay in their homes to help halt the chain of transmission. However, these restrictive measures have undoubtedly affected the social and mental health of individuals (5).

As more and more people are forced to stay at home in self-isolation to prevent the further flow of the pathogen at the societal level, governments must take the necessary measures to provide mental health support as prescribed by the experts (2). The psychological state of an individual that contributes toward the community health varies from person-to-person and depends on his background and professional and social standings (6).

Quarantine and self-isolation can most likely cause a negative impact on one's mental health. A review published in The Lancet stated that the loss of freedom, boredom, separation from loved ones, and uncertainty can cause a deterioration in an individual's mental health (7). To overcome this involves measures at the individual and societal levels to manage the dire situation. Under the current global situation, both children and adults are experiencing a mix of emotions. They can be placed in a situation or an environment that may be new and can be potentially damaging to their health (8).

## Effect on the Children and Teens

Children, away from their school, friends, and colleagues and staying at home can have many questions about the outbreak and they look toward their parents or caregivers to get an appropriate answer. Not all children and parents respond to stress in the same way. Kids can experience anxiety, distress, social isolation, and an abusive environment that can have short or long-term effects on their mental health. Some common changes in children's behavior can be (9).

- Difficulties with concentration and attention
- Changes in eating habits
- Excessive crying and annoying behavior
- Increased sadness, depression, or worry
- Unexpected headaches and pain throughout their bodies
- Changes in or avoiding activities that they enjoyed in the past.

Helping to offset negative behaviors requires that parents remain calm, deal with the situation wisely, and answer all of the child's questions to the best of their abilities. Parents can take some time to talk to their children about the COVID-19 outbreak and share some positive facts, figures, and information. Parents can help to reassure them that they are safe at home and encourage them to engage in some healthy activities including indoor sports and some physical and or mental exercises. Parents can also develop a home schedule that can help the children to keep up with their studies. Parents should show less stress or anxiety at their home as children perceive and feed of the negative energies from their parents. The involvement of parents in healthy activities with their children can help to reduce stress and anxiety and bring relief to the overall situation (10).

## Effect on the Elderly and People With Disabilities

Elderly people are more prone to the COVID-19 outbreak due to both clinical and social reasons such as having a weaker immune system or having underlying health conditions. According to medical experts, people aged 60 or above are more likely to get the SARS-CoV-2 and can develop a serious and life-threatening condition even if they have good health (11).

Physical distancing due to the COVID-19 outbreak can have drastic negative effects on the mental health of the elderly and disabled individuals. Physical isolation at home among family members can put the elderly and disabled person at serious mental health risks. It can cause anxiety and distress and further induce a traumatic situation for them (1). Elderly people depend on young ones for their daily needs and self-isolation can critically damage a family system. Physical distancing does not necessarily mean to refrain from meeting elderly people. COVID-19 can also result in increased stress, anxiety, and depression among elderly people already dealing with mental health issues.

Family members may expect to witness any of the following changes to the behavior of older relatives (12):

- Changes to their sleeping and eating habits
- Irritating and shouting behavior
- Emotional outbursts.

The World Health Organization (WHO) advises and suggests family members to regularly check on older people living within their homes. It is also recommended for them to take a break from the news and social media. Younger family members should take some time to talk to older members of the family and become involved in some of their daily routines if possible (13).

## Effect on Homeless People

The homeless population can experience unique challenges during the COVID-19 outbreak that can affect or further deteriorate their mental health. Homeless people can face the following difficulties, which can exacerbate mental health problems:

- Maintenance of social distance during congregate living
- Availability of proper sanitation facilities
- Less access to healthcare services
- Drug addiction.

Cities with large homeless populations require special arrangements to contain the ongoing outbreaks among the homeless population. Awareness about social distancing, maintenance of cleanliness, timely diagnosis, and adequate healthcare services can help to decrease disease transmission and associated mental health issues (14).

## Effect on People With Mental Health Disorders

Coronavirus SARS-CoV-2 eruption is equally responsible for causing fear, depression, anxiety, and other mental health issues among the mentally challenged individuals in addition to infection. Discrimination, stigmatization, and mental health disorders can increase disease transmission and infection severity among people already dealing with mental health disorders (7). On the 9th of February, 2020, a study reported 50 COVID-19 cases in patients of a psychiatric hospital in Wuhan China. Cognitive impairment, less awareness of the risk and understanding of protective measures, confined psychiatric wards, discrimination, and stigmatization can potentially make treatment less effective (7, 15). The current situation requires the government, healthcare professionals, and the general public to take steps further to curb the transmission of infection (16).

## Effect on Healthcare Workers

Doctors, nurses, and paramedics working as a front-line force to fight the prevailing outbreak are more prone to develop mental health symptoms. Fear of catching a disease, long working hours, unavailability of protective gears and supplies, patients load, unavailability of proper COVID-19 medication, death of their colleagues (after exposure to COVID-19), social distancing and isolation from their family and friends, and the patients' dire situation can take a negative toll on mental health (15). The working efficiency of the health workers can change as the pandemic prevails. It requires healthcare workers to take short breaks between their working hours and deal with the situation calmly and in a relaxed manner (6).

## Stigmatization Among the General Community and Healthcare Professionals

Generally, people who are recently released from the quarantine can experience stigmatization and develop a mix of emotions. Everyone can feel differently and can get a different reception by society when they come out of quarantine (17). People who have recently recovered may have to exercise social distancing from their family members, friends, and relatives to ensure their safety. Different age groups take this social behavior differently and it can have both short and long-term effects (3).

Health workers who are trying to save lives and who are fighting on the front lines to protect the public can also experience social distancing, changes in behavior of their family members, and can be stigmatized as being suspected carriers of the disease. They can develop sadness, anger, or frustration because friends or loved ones may have unfounded fears of contracting the disease from contact with their previously infected friend or relative even though they have been determined not to be contagious (6).

However, the current situation requires a clear understanding of the effects of the recent outbreak on the mental health of people of different age groups (18). Spending time with family members, including children and the elderly, involvement in different healthy exercises and sports activities, following a schedule/routine, and taking a break from traditional and social media can all help to overcome mental health issues (7).

## Take-Home Message

- The effects of COVID-19 outbreak on one's mental health is as important as understanding its clinical features, transmission patterns, and management of the outbreak.
- Pandemic dynamics in society alter dramatically with changes in public behavior.
- The present situation requires the instant launch of public awareness campaigns about how to maintain their mental health in the prevailing situation.

## Author Contributions

BJ and AS designed the study. BJ selected and processed the data and wrote the manuscript. ES edited and revised the manuscript. AS and ZM provided useful information. All authors contributed to the subsequent drafts. The authors reviewed and endorsed the final submission.

## Conflict of Interest

The authors declare that the research was conducted in the absence of any commercial or financial relationships that could be construed as a potential conflict of interest.
